# Traversing the multiple nested geographies of NUTs based entrepreneurial ecosystems

**DOI:** 10.1007/s10961-025-10207-9

**Published:** 2025-04-25

**Authors:** Marc Cowling, Ross Brown, Huan Yang

**Affiliations:** 1 Oxford Brookes University, Oxford, UK; 2 University of St Andrews, St Andrews, UK

**Keywords:** Entrepreneurial ecosystems, Spatial unit, Small business, Loan scheme, L26, R11, R12, M21

## Abstract

Entrepreneurial Ecosystems (EEs) have quickly become a key lens for exploring regional entrepreneurial phenomena. Thus far there appears little consensus around the most relevant geographical unit of analysis for examining EEs however, both from a theoretical and an empirical perspective. In this paper, we set out to test whether wider EE geographical units (such as UK regions) have any meaningful relevance to the small firms and their business operations. To address this concern this paper undertakes an empirical analysis of a loan guarantee scheme in the UK, the Recovery Loan Scheme (RLS). Through the empirical lens of the UK SME support scheme, the RLS, we test the relevance of different levels of EE geographical units including NUTS 1, NUTS 2 and NUTS 3. In the case of the UK, which is a diverse collection of nations (England, and three devolved nations, Scotland, Wales, and Northern Ireland), we found that the three devolved nations, and also London shows much larger and stronger higher order spatial effects on their lower order constituent spatial levels. This suggests that outside of London, and the devolved nations, simply analysing NUTS 1 regions does not appear to be the appropriate level if we want to understand the inherent spatial dynamics of small firm ecosystems. Rather, we need to go to smaller spatial levels to establish the true nature of the ecosystem relevant to the small firm. The policy implications point toward the need for properly tailored and localised policy formulation.

## Introduction

The literature on entrepreneurial ecosystems (henceforth EEs) continues to grow at a feverish pace with little signs of this abating (Alvedalen & Boschma, 2017; Cao & Shi, 2021; Wurth et al., 2022).^1^ The strong traction the concept has gained is testament to the richness and deep-seated complexity inherent in territorial economies combined with their intricate and ever-evolving underlying entrepreneurial micro-foundations (Brown & Mason, 2017; Brown et al., 2023; Spigel & Harrison, 2018). Somewhat ironically however, especially given the spatial antecedents of the concept (Malecki, 2018), a somewhat neglected aspect of this extant literature is an explicit focus on the most appropriate spatial “unit of analysis” which should be adopted when empirically delineating these complex entrepreneurial phenomena (Fischer et al., 2022; Perugini, 2023; Schäfer et al., 2024). Indeed, some bemoan the fact it is “not always clear” what unit of analysis is adopted in terms of unpacking the “regional dimension” of EEs (Coad & Srhoj, 2023, p. 8). This paper therefore wishes to directly address some of these issues by exploring the multi-scaler nature of EEs and how their economic geographies are nested within wider territorial jurisdictions.

Scholars have adopted a variety of spatial scales to empirically examine EEs (Fischer et al., 2022). A prominent way of spatially analysing EEs is to use existing standard statistical jurisdictions such as the Nomenclature of territorial units for statistics, abbreviated NUTS (from French *Nomenclature des Unités Territoriales Statistiques*). This is a geographical nomenclature subdividing the economic territory of the European Union (EU) into regions at three different levels, moving from larger to smaller territorial units i.e. NUTS 1, 2 and 3 respectively (Stanickova & Melecký, 2018). This has been a common methodological approach used within the literature in a number of different studies on EEs using NUTS 2 and 3 levels (see Friesenbichler & Hölzl, 2020; Perugini, 2023). Some hold the view that while the ideal unit of analysis should be relatively disaggregated (Andrews et al., 2022; Coad & Srhoj, 2023; Leendertse et al., 2022) due to data unavailability quantitative research on EEs has traditionally tended to view (and compare) these phenomena at a fairly aggregated (i.e. NUTS 1 and 2) levels (see for example, Bruns et al., 2017; Content et al., 2020; Stam and Van de Ven, 2021). In this paper we wish to posit that the most relevant spatial analytical units tend to be more finely disaggregated ones which can better capture the specifics of any given locational context (i.e. NUTS 3). This is now becoming increasingly recognised in current research on EEs which commonly utilises NUTS 3 as the spatial unit of analysis in studies (see, for example, Audretsch & Belitski, 2021; Cavallo et al., 2023) which owes to the fact that this unit of analysis is typically “characterized by the presence of frequent economic interactions” (Cavallo et al., 2023, p. 1865).

Plus, to date empirical research has largely adopted a singular spatial unit of analysis to empirically unpack certain facets of different EEs. A common focus has been to examine the preponderance and longevity of high growth firms and or levels of start-up rates at different spatial levels of analysis such as NUTS 2 (Leendertse et al., 2022) and NUTS 3 (Coad & Srhoj, 2023; Friesenbichler & Hölzl, 2020). While this yields important comparative insights, how different spatial units of analysis interact is often overlooked and fails to illustrate the muti-scaler nature of how EEs play out on a number of multiple spatial levels. Some authors believe that although some level of spatial unit needs to be chosen for research purposes, multi-level methods are often the most appropriate ones for explaining entrepreneurship when considering sub-national spatial units (Perugini, 2023).

Small and medium sized enterprises (SMEs) tend to be strongly rooted in the circumstances in local economies (Gries & Naudé, 2009), so the use of very large spatial administrative units like NUTS 1 and 2 may be questionable given the inherent heterogeneity across such large territorial jurisdictions. It appears that the lower the spatial level of analysis adopted the more granular the picture of entrepreneurial phenomena emerges (Andrews et al., 2022; Leendertse et al., 2022; Perugini, 2023). This is particularly salient from a policy perspective. It is fair to say, that UK policymakers have taken a rather confusing and often contradictory view on which spatial level is best to support new and smaller firms (Martin et al., 2022). Thus, we have cycled from the region as a focal point, to the sub-regional level, and now multiple regions combined through supra-regional agencies and investment funds such as the so-called Northern Powerhouse region which encompasses a full NUTS 1 region (Lee, 2017; Parr, 2017). While generic types of transactional policy support for standardised assistance for things like grants can be administered at higher order spatial levels such as NUTS 1, when policies are highly tailored and firm specific (like financial support or business advice) a lower order spatial form of delivery mechanism seems vitally important. This is because as empirical findings reveal EEs are highly “spatially heterogeneous and therefore targeted interventions are essential to foster and stimulate the conditions” that shape particular EEs at sub-national and/or provincial level (Perugini, 2023, p. 242). This corresponds with the strong belief that proper customisation of policy directed towards EEs is crucial because entrepreneurial mechanisms take a different form in different local places (Brown & Mason, 2017; Ortega-Argilés, 2022).

Ostensibly, the main factor behind the focus on singular spatial units of analysis owes to the adoption of a mono-scaler lens within most of the work on EEs. According to Brown et al (2025) and others (Fischer et al., 2022; Theodoraki & Catanzaro, 2022), by far the most dominant focal point for research on EE has been a mono-scaler perspective. Typically, EEs are viewed in isolation rather than being “nested” withing wider economic geographies connected via a range of local, regional, national and international resource configurations (Spigel, 2022; Burström et al., 2024). Empirical studies have concentrated on the geographically bounded nature of different endogenous actors and how they mediate the performance of the local EEs. Brown et al (2025) maintain that this mono-scaler focus on analysing spatial locations in isolation is now increasingly being called into question by scholars. Arguably, far from being disconnected from other spatial areas, EEs are increasingly being viewed from a multi-scaler theoretical vantage point in order to fully capture the full complexity of how space shapes entrepreneurial activity and opportunity (Schäfer et al., 2024; Theodoraki et al., 2023).

This paper therefore seeks to answer the following research question: *what is the most relevant spatial scale (such as, NUTS 1, NUTS 2 and NUTS 3) for analysing how EEs function and operate in terms of the behaviour of their entrepreneurial constituents such as SMEs*? This paper seeks to empirically unpack the spatial inter-relationships between these EEs by looking at the spatial distribution of small business support under the Recovery Loan Scheme (RLS) which is a flagship government-backed loan scheme designed to support access to finance for UK SMEs as they look to invest and grow.^2^ This scheme superseded the Enterprise Finance Guarantee which ran from 2009–2020 and Covid-19 related loan guarantee schemes which were terminated in the post-pandemic era (Cowling et al., 2024a, 2024b). RLS supports a wide range of products, covering term loans, overdrafts, asset finance and invoice finance facilities^3^ and firms can borrow up to a maximum of £2 million per business group. Repayment terms are up to six years (for term loans and asset finance) and up to three years (for overdrafts and invoice finance facilities). The scheme is viewed as a pivotal mechanism for injecting business confidence and growth into the UK economy in the wake of the post-pandemic recessionary period.^4^

The paper sets out as follows. First, we assess the relevant literature on EEs. Second, the definitions, data and descriptive statistics are outlined. Third, the results from our models are presented. Fourth, we discuss the relevance of these findings for the EE literature and policy implications thereof. Finally, we end with some brief conclusions.

## Entrepreneurial ecosystems: definitional and conceptual limitations

The literature increasingly conceives entrepreneurship to be a collective endeavour rather than individual undertaking (Hruskova, 2024) which helps explain why the systemic concept of ecosystems has gained such strong traction with scholars and policy makers alike in recent years (Spigel & Harrison, 2018). While this perspective has been universally recognised as having significant academic merit (Brown et al., 2023; Wurth et al., 2022), consensus around a unified definition is largely absent in the literature on EEs. While different definitions abound (Rocha et a, 2022; Cavallo et al., 2019), probably one of the areas obtaining the least level of concurrence concerns the most appropriate spatial parameters demarcating EEs (Credit et al., 2018; Malecki, 2018). How to best delineate EEs as a spatial unit of analysis has been largely side-stepped by scholars (Schäfer et al., 2024) with some claiming rather vaguely they could be a “city, a region or a country” (Stam, 2015, p. 1764). This wide definitional latitude has understandably led scholars to poignantly pose the question: “at what scale should this concept be operationalized” (Credit et al., 2018, p. 11).

This ambiguity perhaps stems to the indeterminate manner of their geographical boundaries which has tended to go somewhat overlooked thus far in the literature (Fischer, 2022; Schäfer et al., 2024). Indeed, some pay lip service to the spatiality of EEs by viewing them simply as “the union of *localized* cultural outlooks, social networks, investment capital, universities, and active economic policies” (Spigel, 2017, p. 49). Others take EEs to mean a “set of interdependent actors and factors coordinated in such a way that they enable productive entrepreneurship within a particular *territory*” (Stam, 2015, p. 1765). One of the most expansive and widely adopted definitions views EEs as a set of interconnected entrepreneurial actors, entrepreneurial organizations, institutions and entrepreneurial processes “which formally and informally coalesce to connect, mediate and govern the performance within the *local* entrepreneurial environment” (Mason & Brown, 2014 p. 5). Again, this hints that EEs are an innately localised phenomenon, albeit a somewhat spatially underspecified one. While clearly at the core of the concept is the view “geography matters”, perhaps in the rush to deploy the concept there is a need for more “systematic scrutiny” around their geographical scope and spatiality (Schäfer et al., 2024, p. 2).

Relatedly, the conceptual lens through which EEs have been viewed may muddy these (already) murky waters further. EE research is commonly viewed as being largely atheoretical (Harrison and Spigel, 2018; Cao & Shi, 2021) and empirically conveyed as a mono-scaler phenomenon (Brown et al., 2025). Indicative of this is the fact that EEs are typically viewed in isolation rather than something which is superimposed in wider economic geographies via a range of endogenous and exogenous resource flows and actors (Schäfer et al., 2024). Empirical studies typically have concentrated on the geographically bounded nature of different endogenous actors and how they mediate the performance of local EEs. This reveals itself in the fact that most studies use fixed administrative units such as cities or local authorities as the main analytical unit of analysis when examining EEs (Credit et al., 2018; Fischer et al., 2022). An example of this is the dominant tendency to use self-contained standardised data such as the NUTS nomenclature for entrepreneurial phenomena such as start-up levels, attitudes towards entrepreneurship, entrepreneurial finance and so on (see, for example, Leendertse et al., 2022). Arguably EE research has been hamstrung by being somewhat data-led in this respect.

However, this mono-scaler lens is now increasingly being called into question with scholars keen to sketch out their spatial boundaries more precisely (Fischer et al., 2022; Theodoraki & Catanzaro, 2022; Brown et al., 2025). In contrast to this narrow viewpoint, others portray the complex nature of EE as a “multi-level, multi-scalar, multi-modal and multi-nodal concept” (Theodoraki et al., 2023, p. 13). Relatedly, some view EEs as resembling a “Russian doll” in that local areas are nested in the regions, which are in turn nested in nations, which are then nested within supranational contexts (Wurth, 2022). In sum, the geographical boundaries of EEs are likely to have a much more complex spatial architecture than has been previously conceptualised in the literature which often views EEs reductively as detached territorial “islands”. To help develop how locations are best analytically observed, arguably a wider lens is required. EEs are perhaps more appropriately conceived as existing together within a wider set of territorial jurisdictions -analogous to overlapping circles in a Venn diagram- rather than self-detached disembodied spatial entities.

## Definitions, data and descriptive statistics

### Definitions

The Nomenclature of territorial units for statistics, abbreviated NUTS (from French *Nomenclature des Unités Territoriales Statistiques*) is a geographical nomenclature subdividing the economic territory of the European Union (EU) into regions at three different levels (NUTS 1, 2 and 3 respectively, moving from larger to smaller territorial units).^5^ Above NUTS 1, there is the ‘national’ level of the Member States. The NUTS is based on Regulation (EC) No 1059/2003 of the European Parliament and of the Council of 26 May 2003 on the establishment of a common classification of territorial units for statistics (NUTS), which is regularly updated.

- NUTS1: Major socio-economic regions (one NUTS1 area typically contains several NUTS2 areas);
- NUTS2: Basic regions for the application of regional policies (one NUTS2 area typically contains several NUTS3 areas);
- NUTS3: Small regions for specific diagnoses.

In keep.eu, NUTS 0 is used to refer to countries for user convenience, even though the NUTS 0 level is not recognized as such by Eurostat.

To give an example from our UK data: The UK is the NUTS 0 spatial unit. Scotland is a NUTS 1 region located within the UK NUTS 0 spatial unit. Eastern Scotland is a NUTS 2 region embedded in Scotland and the wider UK. Falkirk is a NUTS 3 region embedded in Eastern Scotland, Scotland, and the UK. Falkirk had a human population of 165,340 in 2023, Eastern Scotland had a population of 2,049,800, and Scotland had a total population of 5.4 million people, out of a total UK population of 67.74 million people.

It is interesting that the incumbent UK government from 2010 to July 2024 has pursued an industrial strategy that has led to the creation of super regions above NUTS 1 level since 2016. For example, the Northern Powerhouse (NP) forms part of the government’s industrial strategy with the aim of helping businesses up and down the country seize the opportunities presented by leaving the EU (Lee, 2017; Parr, 2017). The Northern Powerhouse geography covers all 11 Northern Local Enterprise Partnerships (LEP) areas as well as North Wales.^6^ The government regards the NP area as comprising the NUTS-1 territories of the North West, Yorkshire and the Humber, and the North East (Parr, 2017). Mackinnon (2020, p. 613) refers to this as a ‘state spatial strategy’ that was ‘undermined by a lack of government commitment and investment following the Brexit vote of 2016’. We also note that the same government also ended funding for the 36 Local Enterprise Partnerships in April 2024 and their functions were to be integrated into Local Authorities which is a very low level of political spatial unit in the UK. Thus, we have a clear tension and confusion in terms of spatial economic investment by the public sector into these super regions (which might fit into the NUTS 0.5 nomenclature if it existed) and local economic strategic planning by Local Authorities (which are more like a NUTS 2 or 3 spatial unit).

### Data and descriptive statistics

To unpack the complex multi-scaler nature of EEs we view this phenomenon via a multi-scaler lens using different levels of NUTS administrative categories in the UK. This paper seeks to empirically unpack these inter-relationships by looking at the spatial distribution of small business support under the RLS which is a flagship government-backed loan scheme designed to support access to finance for UK SMEs as they look to invest and grow.^7^ Data for the RLS covers the period between 2021 and 2024.^8^ This loan guarantee scheme superseded the Enterprise Finance Guarantee which ran from 2009–2020 and Covid-19 related loan guarantee schemes which were terminated in the post-pandemic era (Cowling et al., 2024a, 2024b). RLS supports a wide range of products, covering term loans, overdrafts, asset finance and invoice finance facilities^9^ and firms can borrow up to a maximum of £2 million per business group. Repayment terms are up to six years (for term loans and asset finance) and up to three years (for overdrafts and invoice finance facilities). The scheme is viewed as a pivotal mechanism for injecting business confidence and growth into the UK economy in the wake of the post-pandemic recessionary period.

**Fig. 1:**
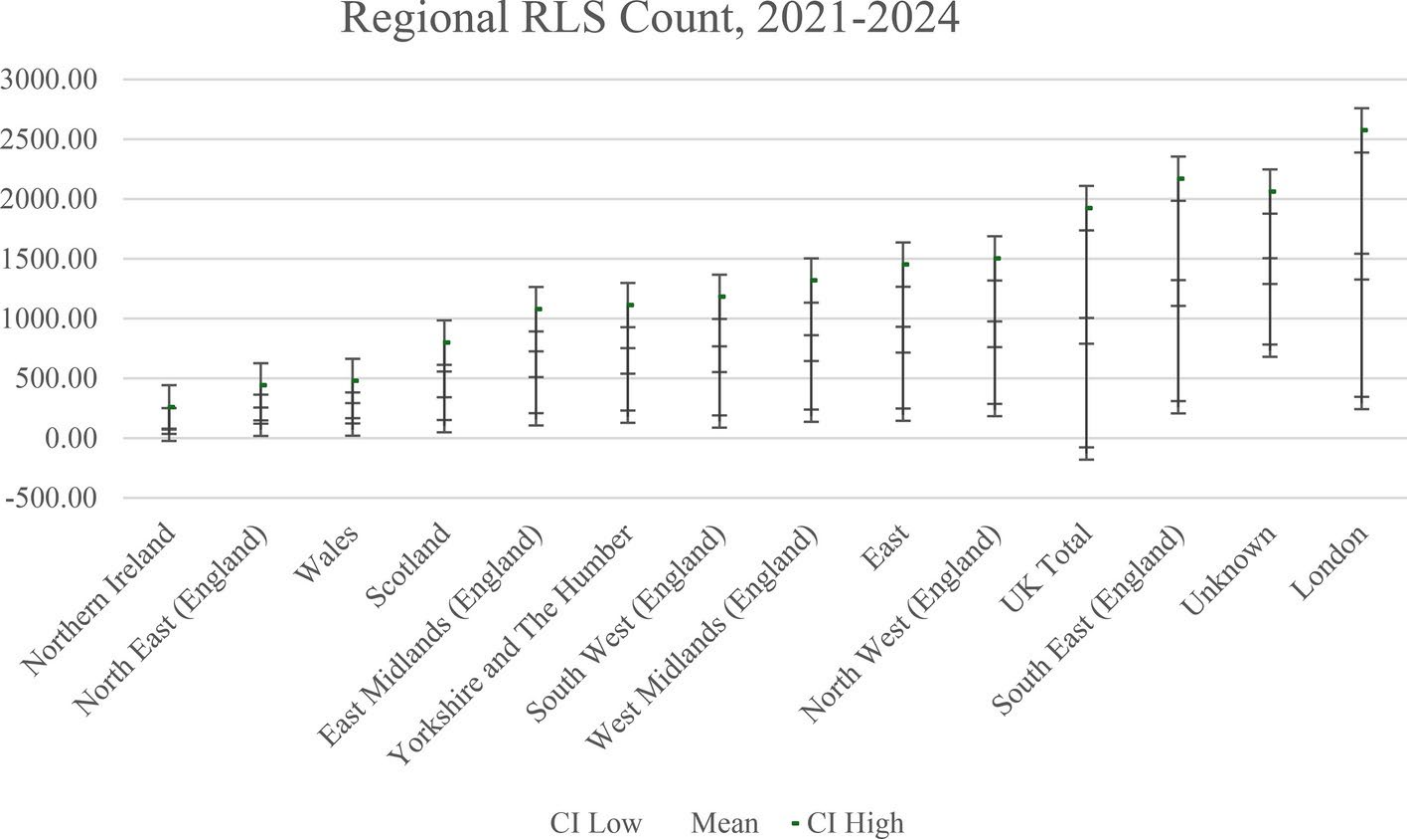
Regional RLS count, 2021–2024. *Notes* Unknown is where RLS loan firm has an unidentified NUTS 1 region

From Fig. 1, we observe that the NUTS 1 regional counts for the whole UK averages 897.21 firms (or loans) and has a wide 95% confidence interval. Across the NUTS 1 regions of the UK, RLS counts are significantly different (Bartlett’s Test of Equal Variances, χ2 = 1.2e + 04, Prob > χ2 = 0.0001. However, in respect of being outside the lower CI bound of other NUTS 1 regions, only Northern Ireland (upper CI bound) is below the lower CI bounds of South East England and London. If we simply looked at differences in mean RLS counts, then South East England (1,213.95) and London (1,434.02) are higher than all other regions, and North West England (868.84), East of England (823.84), and West Midlands of England (753.19) are above the average for Northern Ireland (142.23), North East England (256.23), Wales (275.15), and Scotland (449.94). This would be a cause for consternation amongst policy-makers in the devolved parliaments and assemblies of the non-English nations of the UK if they didn’t consider the confidence intervals for each NUTS1 region RLS counts. In fact, a stronger case for NUTS 1 level spatial ‘unfairness or inequality’ would be around the deficit compared to London and the South East of England by policy-makers in Northern Ireland.

**Fig. 2:**
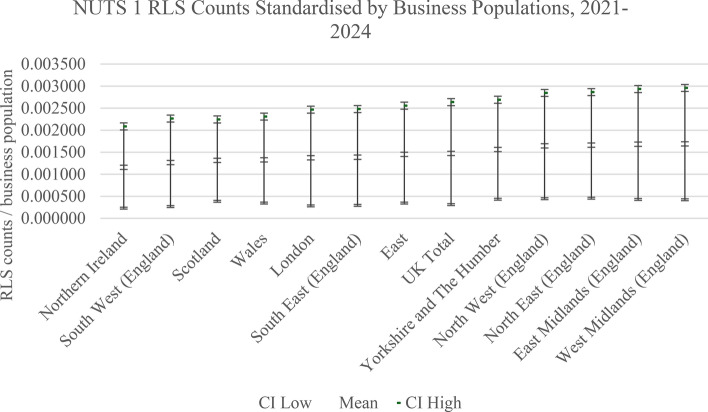
RLS counts adjusted for the NUTS 1 business populations

From Fig. 2, which standardised the NUTS 1 level RLS counts by the NUTS 1 business populations, we observe that the UK average is 0.147% of the business population accessing an RLS loan. After standardisation, we observe that the NUTS 1 regions with a high relative share of RLS has changed significantly with the highest relative shares in West Midlands (0.169%), East Midlands (0.168%), North East (0.166%), and North West (0.156%), and low relative shares in Northern Ireland (0.116%), South West (0.127%), Scotland (0.132%), Wales (0.133%), London (0.137%), and South East (0.139%). However, it is evident that, whilst the NUTS 1 variances at first glance appear quite modest (from a low of 0.116% to a high of 0.169%) are statistically significant (Bartlett’s Test of Equal Variances, χ2 = 364.238, Prob > χ2 = 0.0001). However, as we can visually observe, the lower CI bound for West Midlands never goes outside of the 95% CI for Northern Ireland.

Whilst it remains the case that Northern Ireland appears to be at a general absolute disadvantage in terms of its RLS count and standardised shares of RLS loans, and to a lesser degree also Scotland and Wales, the precise nature of this disadvantage is different if we compare high RLS count NUTS 1 regions and high relative RLS shares. On the former London and the South East appear disproportionately advantaged and, on the latter, it is the West Midlands, East Midlands, and North East. The North East region is particularly interesting as it moves from second lowest on RLS counts to third highest when we standardise for the NUTS 1 business population.

In general, using absolute counts and relative shares standardised by the respective NUTS 1 business populations, we observe that the variances and 95% CI are fairly large. This raises the question of whether NUTS 1 regions are the appropriate unit of analysis or whether we need to consider lower-level spatial units such as NUTS 2 and NUTS 3. This is particularly relevant as we are investigating a small firm targeted policy directly after the Covid-19 pandemic which is intended to promote access to capital for growth as firms emerge from the pandemic laden with debt, the debt overhang problem, and with low residual cash-flows. During the pandemic period more than 1 m UK SME accessed government supported loan guarantee schemes (Calabrese et al., 2022) and default rates were non-trivial (Cowling et al., 2024a, 2024b), although there was clear evidence that these schemes were spatially equitable in their relative distribution across UK NUTS 1 regions (Cowling et al., 2023).

The key point more generally is that the overwhelming majority of small firms are rooted in their immediate locality in the sense that they use local resources (labour and materials) and trade with local consumers (Cowling & Nadeem, 2020). This suggests that if spatial ecosystems are to be meaningful, then it is less likely to be apparent at the NUTS 1 level. For example, the South East NUTS 1 region of the UK has a land area of 19,072 square kilometres. From Hastings in the south-east of the region to Cherwell in the north west of the region is 210.5 km. Whether a small local butcher in Hastings would even consider whether there was a unique resource available in Cherwell within the wider South East NUTS 1 region is debatable. However, they might consider the availability of a unique resource in their NUTS 3 area which is West Sussex (South West) or the spatially proximate NUTS 3 area of West Sussex (North East), or even the wider NUTS 2 area of Surrey, East and West Sussex.

As an illustrative example, moving down from NUTS 1 to NUTS 2, and finally to NUTS 3 spatial levels uncovers some important spatial variations, we use Scotland (NUTS 1 region and a country with a devolved parliament) as our case study (see Appendix Table 5). At NUTS 1 level, Scotland has a UK share of 5.46% of RLS loans. Its relative share of the 2021 UK total business population is 5.69%, total UK population is 5.5%, and GDP is 7.42%. In these respects, its NUTS 1 RLS share is fairly proportional to its business population share and human population, although below its relative share of UK GDP.

Within the NUTS 1 region of Scotland, if we move to the NUTS 2 level, then we observe some non-trivial variations in RLS counts. Eastern Scotland has the highest relative share of Scottish RLS counts at 36.16% and West Central Scotland has a 28.16% share. In contrast, the Highlands and Islands share is relatively low at 8.36% as is that of North Eastern Scotland (11.48%), and Southern Scotland (15.40%). Whilst the difference between RLS counts for Scotland and the highest NUTS 1 region of London is large with a factor of 1.99 times, the difference between the lowest NUTS 2 Scottish area (Highlands and Islands) and the highest (Eastern Scotland) is even larger at a factor of 3.33 times. In this sense, within Scotland NUTS 2 variations in RLS counts are materially larger than that between Scotland and London NUTS 1 variations.

If we move to the lowest spatial ordering which is NUTS 3 then it becomes apparent that there is a further source of variation that gets down to the very local level. In the NUTS area of Eastern Scotland, the City of Edinburgh has a share of 30.30% and this compares to 6.54% in West Lothian. In the Highlands and Islands, the highest share is in Inverness and Nairn, Moray and Badenoch and Strathspey at 38.62% compared to 4.83% in Na h-Eileanan Siar. In Southern Scotland, the highest share is in South Lanarkshire at 41.95% and the lowest in the Scottish Borders at 11.24%. Finally, in West Central Scotland, the highest share is recorded for Glasgow City at 52.42% and the lowest for East Dunbartonshire, West Dunbartonshire, and Helensburgh and Lomond at 8.27%. The within NUTS 2 area variation across NUTS 3 areas is very large and indeed much larger than within a NUTS 1 region across NUTS 2 areas.

This evidence raises the big question of whether NUTS 2 and NUTS 3 regions have any systematic relationship with the broader NUTS 1 region. The study of spatial contagion in a small business context has revealed some important findings, although often this has been in the context of business failure. For example, in their research on small Spanish hotels, research found that the (failure) contagion effect is significant and exerts different effects over time (Vivel-Búa & Lado-Sestayo, 2023). Calabrese (2023), in her study of spatial contagion and small business failure in London, found that contagion manifests itself within spatially clustered industry sectors rather than in the wider population of small businesses per se. This is a particularly important and nuanced finding as it suggests that closely linked firms (i.e. within an industry cluster) should be the point of focus if we are considering spatial contagion in ecosystems. It also suggests that the appropriate spatial unit is not NUTS 1 but a lower level of spatial unit.

**Fig. 3:**
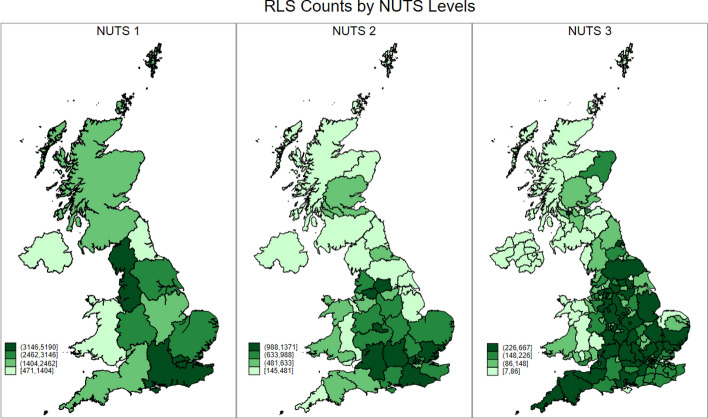
RLS counts by NUTS levels

In our context, which is credit rationed small firms emerging from the Covid-19 pandemic and who accessed an RLS guaranteed loan, these failure contagion effects can be considered as the ultimate outcome of a small firm being credit rationed. This under-capitalisation can ultimately lead to the firm's demise as it runs out of cash and becomes increasingly uncompetitive (Cowling et al., 2020a, 2020b; Dörr et al., 2022) and this process has been shown to feature significant lower order (i.e. NUTS 3) spatial variation (Brown & Cowling, 2021; Cowling et al., 2024a, 2024b). Figure 3 maps the RLS counts at all three NUTS levels, which also show such significant lower order spatial variation in RLS counts.

It follows that credit rationing per se, and particularly after the pandemic, has a distinct industry component as not all industry sectors faced strict lockdowns during the pandemic period, but also more generally as firms in different industry sectors face differences in the volatility of their cash-flows and the availability of collateral (Bonnet et al., 2016; Brown et al., 2020; Drakos & Giannakopoulos, 2011). In many countries the hospitality sector was locked down completely during the pandemic, with agriculture often being a protected industry sector that remained under business-as-usual conditions (Di Porto et al., 2022; Busato et al., 2021; Faber et al., 2020).

Whilst the presence of broader credit rationing amongst smaller firms and how spatial ecosystems impact on it is important, it is equally important to understand the context of any credit rationing. For example, after the pandemic period there may be emergent signs of economic growth and to take advantage of this smaller firms may need to invest in growth capacity to meet this new demand for their products and services. It may also be the case that many smaller firms have not recovered from the crisis in terms of being able to rebuild their operating cash which is a liquidity problem. These firms will have a demand for short-term lending to support day-to-day operational needs such as paying wages and buying raw materials. Other firms, with significant pre-existing debts may seek to refinance these debts on more favourable terms. All of these types of small firms may face a debt overhang problem when seeking conventional bank loans, hence the transition from the large and generous Covid-19 public loan guarantee schemes into the post-pandemic period public loan guarantee schemes of which, in the UK, the RLS is the flagship programme. We note that the debt overhang problem was identified during the Global Financial Crisis period (Botta, 2020; Vanlaer et al., 2021) and is also a significant concern for the OECD currently (Demmou et al., 2021).

**Fig. 4:**
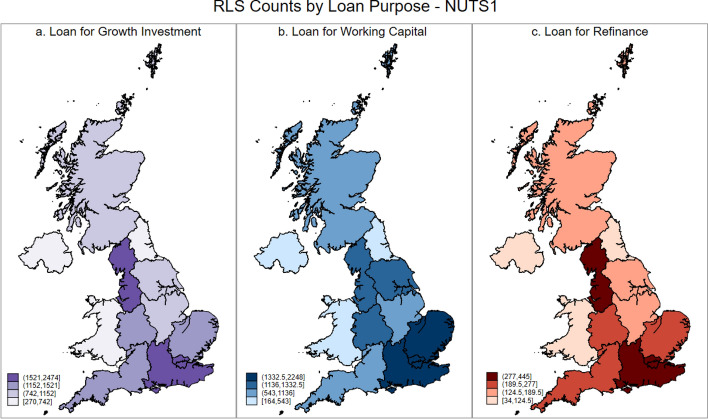
RLS counts by loan purpose at NUTS 1 level

**Fig. 5:**
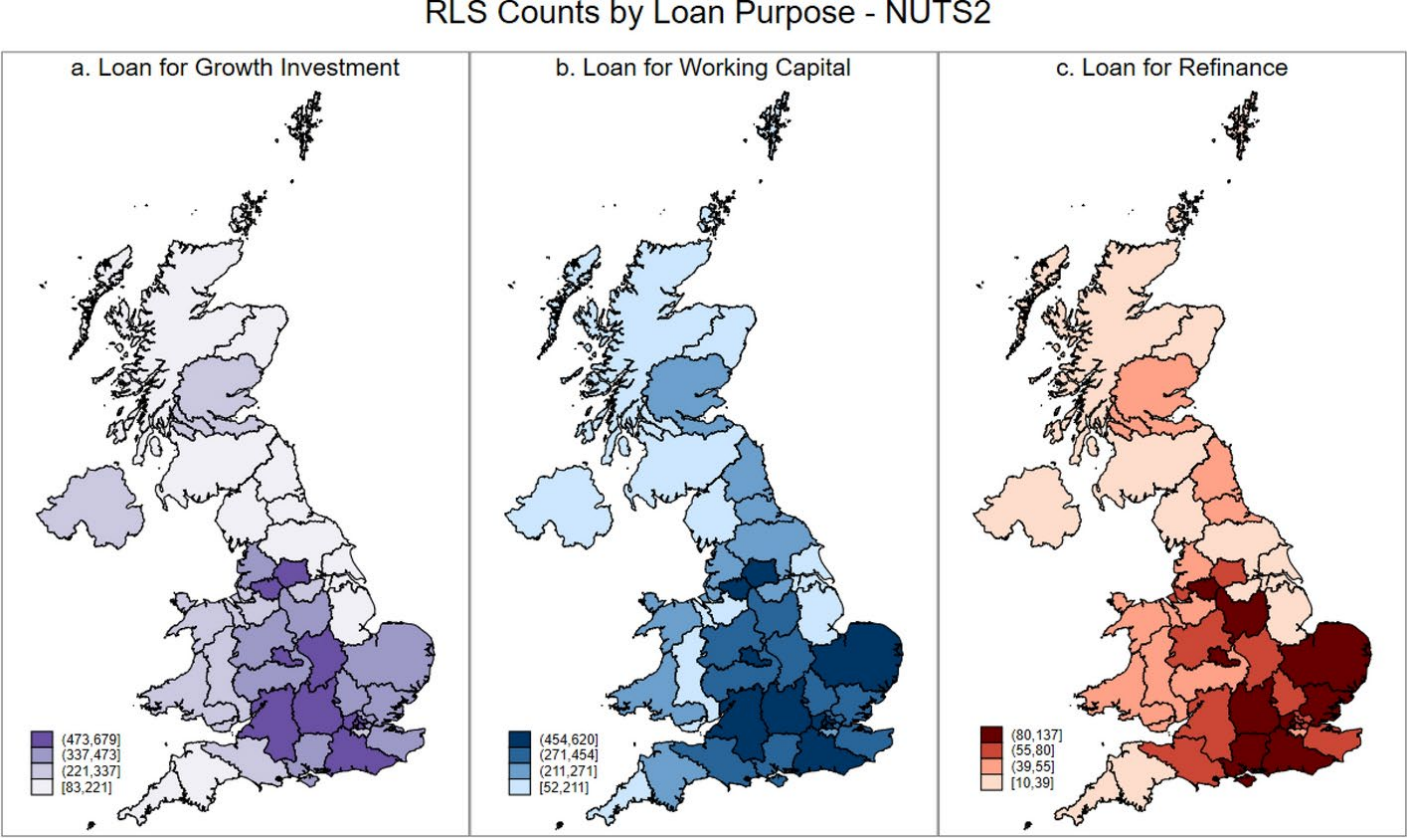
RLS counts by loan purpose at NUTS 2 level

**Fig. 6:**
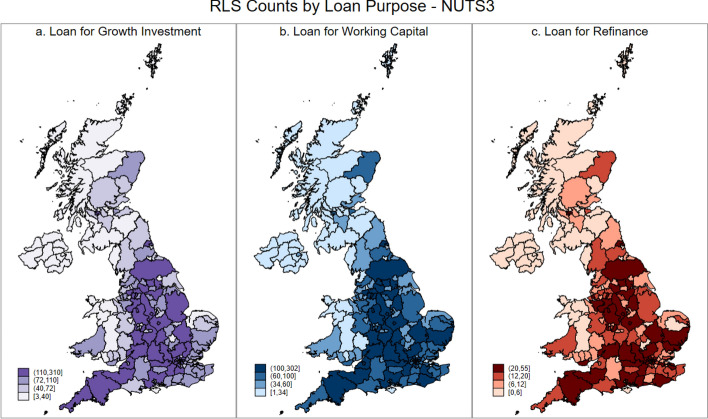
RLS counts by loan purpose at NUTS 3 level

As general credit rationing of smaller firms has distinct spatial dynamics, it follows that spatial differences will also manifest themselves if we consider the precise nature of the credit rationing that small firms face. We address this by considering, at the NUTS 1, NUTS 2, and NUTS 3 levels, RLS use for three distinct purposes: growth related investment; working capital, and; refinancing existing debts. These data are presented in the UK maps below, where Figs. 4, 5 and 6 presents the maps for RLS counts by loan purposes at NUTS 1, 2 and 3 Level, respectively.

## Modelling RLS loan counts at different spatial levels

We begin by modelling RLS counts at the NUTS 1 level. This provides a baseline that we can compare our lower order NUTS 2 and NUTS 3 spatial levels against. As we are specifically interested in what is the most appropriate spatial level to understand how ecosystems impact on credit rationing of smaller firms, we include the NUTS 1 region in the NUTS 2 count model and the NUTS 1 and NUTS 2 spatial units in the NUTS 3 model. If the most appropriate spatial level for understanding how ecosystems impact on credit rationing of smaller firms and the demand for public loan guarantees is NUTS 1, then we would expect to see large and significant effects in the NUTS 2 and NUTS 3 count models. If the NUTS 2 effects were larger than the NUTS 1 effects in the NUTS 3 count models then we would take this as evidence that the spatial level for understanding ecosystems in the context of small firms is below NUTS 1.

### Poisson count models

Our first set of models are count models for the number of firms within a defined spatial unit, $Y=RLS Counts$, and is estimated by a poisson model of the form^10^:

$$P\left(Y=y|\lambda \right)=\frac{{e}^{-\lambda }{\lambda }^{y}}{y!}$$

$$\lambda =\mathrm{exp}\{X\beta \}$$

For the set of explanatory variables $X$, we include the NUTS 1 or NUTS 2 spatial units as discussed earlier. As our literature review also suggested that industry effects were also important in understanding credit rationing and spatial outcomes, we also include a full set of industry sector dummy variables in each model. We also include firm size and age as both have been identified as central to small firm credit rationing and economic outcomes.

Table 1 reports the results for three alternative models including [1] the effect of NUTS 1 on NUTS 3 counts, [2] the effect of NUTS 2 on NUTS 3 counts, and [3] the effect of NUTS 1 on NUTS 2 counts. If the lower order (NUTS 2) effects on the lowest order of spatial unit (NUTS 3) is greater or explains more of the variance, than the higher order spatial unit (NUTS 1) does, then this suggests that the most appropriate level for examining a spatial ecosystem is at smaller levels of spatial unit than the region (or NUTS 1) level. Equally, if the highest spatial unit, NUTS 1, explains more of the variation in NUTS 2 counts than NUTS 3 counts, then the regional effect is weaker and less representative of an ecosystem that is relevant to smaller firms who are rooted in their local areas where they trade and access resources from.

**Table 1:** RLS counts base models

Dependent variables:	(1) RLS counts NUTS3		(2) RLS counts NUTS3		(3) RLS counts NUTS2	
	β	OIM S.E	β	OIM S.E	β	OIM S.E
*Key firm characteristics*						
LTD company	0.0149	(0.0148)	0.0098	(0.0145)	0.0120	(0.0124)
Log sales	− 0.0029	(0.0020)	− 0.0045**	(0.0020)	− 0.0017	(0.0017)
Log Age	0.0349***	(0.0035)	0.0363***	(0.0035	0.0255***	(0.0255)
*Loan purpose (base: Growth Investment)*						
Working Capital	0.0284***	(0.0061)	0.0297***	(0.0061)	0.0235***	(0.0053)
Refinance	− 0.0067	(0.0111)	− 0.0040	(0.0111)	− 0.0035	(0.0096)
*Region NUTS1 (base: East of England)*						
East Midlands of England	− 0.0108	(0.0140)			− 0.0256**	(0.0121)
London	0.0414***	(0.0114)			0.0179*	(0.0099)
North East of England	− 0.0770***	(0.0190)			− 0.1393***	(0.0167)
North West of England	− 0.0564***	(0.0128)			− 0.0472***	(0.0110)
Northern Ireland	− 0.4618***	(0.0306)			− 0.1216***	(0.0225)
Scotland	− 0.2260***	(0.0163)			− 0.1849***	(0.0138)
South East of England	0.0044	(0.0118)			0.0225**	(0.0101)
South West of England	− 0.0101	(0.0136)			− 0.0723***	(0.0119)
Unknown	− 0.0022	(0.0488)			− 0.3107***	(0.0486)
Wales	− 0.2259***	(0.0194)			− 0.1196***	(0.0160)
West Midlands of England	0.0152	(0.0130)			− 0.0052	(0.0112)
Yorkshire & Humber	0.0230*	(0.0135)			− 0.0716***	(0.0119)
*Region NUTS2 (base: Bedfordshire and Hertfordshire)*						
Berkshire, Buckinghamshire and Oxfordshire			− 0.0191	(0.0200)		
Cheshire			− 0.1603***	(0.0270)		
Cornwall and Isles of Scilly			− 0.0869***	(0.0336)		
Cumbria			− 0.3460***	(0.0432)		
Derbyshire and Nottinghamshire			− 0.1819***	(0.0224)		
Devon			− 0.1233***	(0.0274)		
Dorset and Somerset			− 0.1294***	(0.0251)		
East Anglia			− 0.1659***	(0.0222)		
East Wales			− 0.2739***	(0.0298)		
East Yorkshire and Northern Lincolnshire			− 0.2924***	(0.0322)		
Eastern Scotland			− 0.3501***	(0.0272)		
Essex			− 0.1709***	(0.0221)		
Gloucestershire, Wiltshire and Bath/Bristol Area			− 0.1237***	(0.0216)		
Greater Manchester			− 0.0722***	(0.0202)		
Hampshire and Isle of Wight			− 0.1757***	(0.0231)		
Herefordshire, Worcestershire and Warwickshire			− 0.0697***	(0.0236)		
Highlands and Islands			− 0.7501***	(0.0591)		
Inner London– East			− 0.0821***	(0.0209)		
Inner London– West			0.0077	(0.0200)		
Kent			− 0.1949***	(0.0236)		
Lancashire			− 0.2830***	(0.0262)		
Leicestershire, Rutland and Northamptonshire			− 0.0745***	(0.0218)		
Lincolnshire			− 0.0837**	(0.0342)		
Merseyside			− 0.2309***	(0.0269)		
North Eastern Scotland			− 0.1538***	(0.0393)		
North Yorkshire			− 0.0792***	(0.0280)		
Northern Ireland			− 0.5729***	(0.0329)		
Northumberland, and Tyne and Wear			− 0.1391***	(0.0276)		
Outer London—East and North East			− 0.1313***	(0.0238)		
Outer London—South			− 0.1454***	(0.0257)		
Outer London—West and North West			− 0.0622***	(0.0203)		
Shropshire and Staffordshire			− 0.0792***	(0.0229)		
South Yorkshire			− 0.0834***	(0.0266)		
Southern Scotland			− 0.5086***	(0.0403)		
Surrey, East and West Sussex			− 0.0991***	(0.0202)		
Tees Valley and Durham			− 0.2384***	(0.0285)		
Unknown			− 0.1124**	(0.0503)		
West Central Scotland			− 0.2239***	(0.0282)		
West Midlands			− 0.1214***	(0.0206)		
West Wales and The Valleys			− 0.3866***	(0.0276)		
West Yorkshire			− 0.0346*	(0.0208)		
Constant	1.2639***	(0.0374)	1.4288***	(0.0397)	1.5782***	(0.0322)
Sector controls	Y		Y		Y	
No. obs	30,149		30,149		30,149	
Model significance	0.00001		0.00001		0.00001	
LR χ2	1,061.65		1,640.42		738.51	
Pseudo R2	0.0103		0.0159		0.0067	

*** Indicates significance at 1% level, ** indicates significance at 5% level, and * indicates significance at 10% level. Figures in parentheses are standard errors

Model [1], which reports the effects of NUTS 1 (regions) on NUTS 3 (the smallest spatial unit) counts, and shows that in six out of twelve regions there is a significant regional effect (or 50.00%). London is the only region where the effect is positive. In contrast, the largest regional effects are identified in Northern Ireland, Scotland, and Wales. These are all regions of the UK with some degree of devolved political and economic power and administration, and Northern Ireland has a unique post-Brexit status due to its land border with the EU through Ireland. The other regions where the magnitude of the effect is much smaller, but still significant, are the North East and North West of England. If we compare this directly with the effects of NUTS 1 on NUTS 2 counts, (Model [3], then we observe a dramatic increase in the number of regions that have significant effects. Here ten out of twelve regions have significant effects. Here, we find that London and the South East of England are the only NUTS 1 regions with a positive and significant effect. Together, these two results suggest that London is a unique ecosystem at all spatial levels of analysis, and that the wider South East region that surrounds it is also important for NUTS 2 ecosystems. The other NUTS 1 regions that have significant, but negative effects on NUTS 2 counts are, in declining order of magnitude of the coefficient effects, include Scotland, North East, Northern Ireland, and Wales, which all have large effects, and South West, Yorkshire and the Humber, North West, and East Midlands, which all have significant effects but much smaller ones. Again, the devolved nations of the UK appear to have stronger and broader ecosystems than England.

However, if we consider the effects of NUTS 2 regions on NUTS 3 counts (model [2]), then we observe that thirty-eight of the NUTS 2 regions (or 90.48%) have a significant effect on the NUTS 3 spatial level. Here, as Northern Ireland has a unique geographical status as being the spatial unit across NUTS 1 and NUTS 2, we are able to directly compare the magnitude of the effects on the NUTS 2 and NUTS 3 spatial levels. On this, we find that the Northern Ireland NUTS 1 on NUTS 3 effect has a $\beta$ of − 0.4618, a $\beta$ of − 0.1216 for NUTS 2 on NUTS 3, and a $\beta$ of − 0.5729 for NUTS 1 on NUTS 2. Thus, the regional effect is largest at higher spatial levels. Other NUTS 2 regions where the effects are large in magnitude on NUTS 3 include the Highlands and Islands of Scotland ($\beta$= -0.7501), Southern Scotland ($\beta$= -0.5086), and West Wales and the Valleys ($\beta$= -0.3866).

Other results from this set of models include significant industry sector effects in models [1], NUTS 1 on NUTS 3, and [2] NUTS 1 on NUTS 2. The largest effects were identified for information and communications, and professional and scientific services, both of which are classified as knowledge intensive services (KIS).^11^ Firm age was also found to be positive and significant across all spatial levels, and firm size was found to be negative and significant, but only in model [2] which was for NUTS 2 effects on NUTS 3 counts. In respect to what firms were using RLS for, we find a positive effect across all models for using loans to fund working capital. In these respects, RLS seems to be supporting older firms in knowledge intensive services across the UK at all spatial levels with working capital. These results suggest that these types of firms are building up financial resources to expand as the economy emerges from the Covid-19 pandemic.

### Nested poisson models

Table 2 uses a nested poisson model specification and allows for firm characteristics (industry sector, limited liability legal status, firm size, and firm age) to enter as block 1, loan purpose (capital expenditure, working capital, and refinancing existing debt) to enter as block 2, and RLS spatial counts to enter as block 3. From this set of models, we find that industry sector variation is only marginally important, and that firm age is only positive and significant in model [2] which is NUTS 2 effects on NUTS 3 counts. Loan purpose is insignificant in all three models. However, we do find that regional counts across all three models are positive and significant. Specifically, we find that the NUTS 1 on NUTS 3 effect has a $\beta$ of 0.2011, a $\beta$ of 0.2115 for NUTS 2 on NUTS 3, and a $\beta$ of 0.1538 for NUTS 1 on NUTS 2. In this sense, the greatest spatial effects occur in respect of NUTS 2 regional RLS counts on NUTS 3 and the weakest effect for NUTS 1 on NUTS 2. This may reflect local knowledge sharing from firms that successfully raised RLS loans with other firms in their spatial proximity. These effects are dissipated at higher order spatial levels.

**Table 2:** RLS counts nested poisson models

Dependent variables:	(1) RLS counts NUTS3		(2) RLS counts NUTS3		(3) RLS counts NUTS2	
	β	OIM S.E	β	OIM S.E	β	OIM S.E
*Key firm characteristics*						
LTD company	0.0117	(0.0145)	0.0124	(0.0145)	0.0048	(0.0124)
Log sales	0.0013	(0.0020)	− 0.0012	(0.0020)	0.0015	(0.0017)
Log Age	0.0062*	(0.0036)	0.0078**	(0.0036)	0.0039	(0.0031)
*Loan purpose (base: Growth Investment)*						
Working Capital	0.0086	(0.0062)	0.0072	(0.0062)	0.0063	(0.0053)
Refinance	− 0.0052	(0.0111)	− 0.0022	(0.0111)	− 0.0028	(0.0096)
Log NUTS1 Count	0.2011***	(0.0042)			0.1538***	(0.0036)
Log NUTS2 Count			0.2115***	(0.0043)		
Constant	− 0.0288	(0.0446)	0.2220***	(0.0415)	0.5791***	(0.0381)
Sector Controls	Y		Y		Y	
No. obs	30,149		30,149		30,149	
Model significance	0.00001		0.00001		0.00001	
LR χ2	2,580.13		240.32		2,065.81	
Pseudo R2	0.0251		0.0263		0.0189	

*** Indicates significance at 1% level, ** indicates significance at 5% level, and * indicates significance at 10% level. Figures in parentheses are standard errors

### RLS counts with major high street bank market share

As a robustness check, we calculated the shares of the major UK high street banks in total lending on the CBILS scheme over the period April 2020 to June 2021 when the RLS replaced the Covid-19 loan guarantee scheme. We choose this scheme as it is most closely related to the RLS scheme in terms of its guarantee coverage rate, loan size, and interest rates within the broader context where 92.1% of total SME lending during the Covid-19 period was under a public loan guarantee scheme (Calabrese et al., 2022). These shares were calculated at the NUTS1 and NUTS 2 levels for the UK. These new spatial variables of major high street bank loan concentration were substituted into our models for RLS counts at NUTS 2 and NUTS3 spatial levels. This provides a direct link between the spatial structure of the SME banking system and the number of firms supported in each spatial area by RLS.

Table 3 presents the results of the models for RLS counts with the major high street bank lending market share at specific spatial levels. Our baseline models for NUTS2 and NUTS3 (columns 1–3) show that the structure of the banking system has positive and significant effects at both spatial levels. Thus, major high street banks share of total loans issued is associated with increases in RLS spatial counts. In the final model (column 4) where we allow for both NUTS1 and NUTS2 banking structures in the determination of NUTS3 counts, we find that both are positive and significant, but the magnitude of the banking structure effects is larger for NUTS2 major high street bank concentration of lending than NUTS1 (β’s = 0.374*** and 0.334***). This suggests that major high street bank presence in the SME loan market increases the total number of RLS loans issued at small spatial levels and that the sub-regional banking structure is more important than the regional banking structure. More generally, it suggests that the major high street banks are attracted to loan guarantee scheme lending as it mitigates the information problems associated with lending distance and transactional lending approaches.

**Table 3:** RLS counts with major high street bank market share

Dependent variables:	(1) RLS counts NUTS3	(2) RLS counts NUTS3	(3) RLS counts NUTS2	(4) RLS counts NUTS3
Major Bank share at NUTS1	0.698*** (0.054)		0.286*** (0.045)	0.334*** (0.097)
Major Bank share at NUTS2		0.583*** (0.045)		0.374*** (0.082)
Sector controls	Y	Y	Y	Y
Loan purpose controls	Y	Y	Y	Y
Firm controls	Y	Y	Y	Y
No. obs	27,178	30,149	27,178	27,178
Model significance	0.0000	0.0000	0.0000	0.0000
LR χ2	390.98	412.26	217.14	411.76
Pseudo R2	0.0042	0.0040	0.0022	0.0044

*** Indicates significance at 1% level, ** indicates significance at 5% level, and * indicates significance at 10% level. Figures in parentheses are standard errors

### Shapley decomposition and total loan cash volume values

A further test of the relative influences of NUTS 1 and NUTS 2 spatial levels on NUTS 3 outcomes was conducted by calculating the Shapley decomposition in Table 4. This decomposition helps us to understand more how each spatial level contributes to a model's predictions. We re-run the original model including NUTS 1 and NUTS 2 spatial classifications in the NUTS 3 level outcomes and calculate the 1st order effects and the Shapley values. We also run the same model but use an additional dependent variable which is the total aggregate cash value of RLS loans at each NUTS 3 level. This gives us a measure of the total spatial variability in credit rationing expressed in cash terms that has been alleviated by the RLS scheme. This is important as it helps us to understand more about how finance constrained firms were and, in the absence of the scheme, how this might have affected the survival and growth of firms at the local level. Aggregate RLS lending at the NUTS 3 level was lowest in Na h-Eileanan Siar, one of the Scottish Islands at £2.26 m and highest in Westminster in the heart of London at £216 m.

**Table 4:** Robustness Test and Shapley Decomposition

Dependent variables:	(1) Log loan counts NUTS3		(2) Log loan size NUTS3		(3) Log loan size NUTS3		(4) Loan default	
		Shapley value		Shapley value		Shapley value		Shapley value
Log NUTS1 Count	0.3634*** (0.0074)	0.2141						
Log NUTS2 Count	0.4866*** (0.0076)	0.3014						
Log Loan Size NUTS1			0.2224*** (0.0077)	0.0530				
Log Loan Size NUTS2			0.5698*** (0.0074)	0.2079				
Major Bank share at NUTS1					− 0.3593*** (0.1352)	0.0381		
Major Bank share at NUTS2					3.5459*** (0.1181)	0.0530		
Loan Default NUTS1							0.0051*** (0.0007)	0.0512
Loan Default NUTS2							0.1652*** (0.0026)	0.1612
Sector controls	Y		Y		Y		Y	
Loan purpose controls	Y		Y		Y		Y	
Firm controls	Y		Y		Y		Y	
Model	Nested OLS		Nested OLS		Nested OLS		Nested OLS	
No. obs	30,149		30,149		27,178		30,149	
R2	0.5314		0.3088		0.1150		0.1927	

*** Indicates significance at 1% level, ** indicates significance at 5% level, and * indicates significance at 10% level. Figures in parentheses are standard errors

The first model, which is for the log of NUTS 3 RLS counts, shows that the 1st order effects are greater in magnitude for NUTS 2 levels (0.6216) than NUTS 1 levels (0.4123) and the Shapley values are 0.3014 and 0.2141 respectively. The second model, which uses our new aggregate cash value of RLS loans at the NUTS 3 level, shows a similar pattern with the 1st order effects being greater in magnitude at the NUTS 2 level (0.2484 compared to 0.0344) and the Shapley values are 0.2079 and 0.0530 respectively. The third model, which uses major high street bank lending concentration as the spatial variable, reports 1st order effects of 0.0693 for the NUTS 2 level and 0.0501 for the NUTS 1 level and Shapley values of 0.0530 and 0.0381 respectively. Thus, across all alternative model specifications the Shapley decomposition shows that the relative importance of NUTS 2 effects on NUTS 3 are greater than the NUTS 1 effects on NUTS 3. Hence, the outcomes at the NUTS 3 levels in terms of RLS counts and the aggregate cash volume of RLS loans are more directly influenced by sub-regional spatial dynamics than regional spatial dynamics.

### Loan default and spatial effects

As a final robustness test, we use RLS loan default as an alternative dependent variable. This helps us to understand more about the potential contribution of RLS lending to spatial economic development through credit rationed firms being able to survive and make an ongoing economic contribution to the local areas they are located in. Areas with a higher survival rate by implication have a greater potential to sustain and create employment and generate positive local economic multiplier effects. The data show that 11.05% (std dev = 31.35) of RLS loans ended in default. The range of default at the NUTS 3 level was large and was lowest at 3.33% in Antrim and Newtownabbey in Northern Ireland and highest in Enfield in London at 21.80%. In terms of default counts at the NUTS 3 level, we find that default was highest in the Hertfordshire County Council district where 98 RLS loans ended in default.

Our results on default counts at the NUTS 3 level using the nested form of the model shows that NUTS 2 default counts have a greater effect on NUTS 3 default counts than the corresponding NUTS 1 default counts. The Shapley decomposition shows that the 1st order effects are greater in magnitude for NUTS 2 levels (0.2447) than NUTS 1 levels (0.1313) and the Shapley values are 0.1612 and 0.0530 respectively. Thus, even if we change the dependent variable to account for loan default, the general finding that sub-regions influence district level outcomes more than wider regional effects hold. This strand of analysis shows that the ability of small firms to survive within a local district ecosystem varies significantly across the districts of the UK and hence the potential for firms to make a sustained economic contribution to their districts will be different.

## Discussion

Within this paper we set out to consider at what spatial level we should consider EEs that are most relevant to smaller firms. This work makes a number of important contributions both on a theoretical and policy level. In terms of the latter, at the EU level and in the UK, the focus is very much on regions, or in the UK’s case increasingly super-regions such as the Northern Powerhousee as the spatial unit for the design of policy and the distribution of SME support funding (Lee, 2017).^12^ The reason for questioning the use of NUTS 1 regions as the focus for policy making is that most small firms trade within small, geographically localised markets, and they also access their resource inputs from their local markets (Cowling & Nadeem, 2020; Gries & Naudé, 2009). In other words, small close-knit economies are relatively self-contained. In this respect it is an empirical and economically important question as to whether the wider region, which may extend for a hundred miles away from a small firm's shop, office, or factory has any meaningful relevance to them and their business operation.

The lens we use is the loan market as access to loan funds is key for the survival and growth of smaller firms, and one in which they typically find the most challenging for a number of reasons including their size per se, and the relative informational opacity (Berger & Udell, 1998; Cassar, 2004). The UK was relatively unique until very recently as it had generally implemented nationally available SME support schemes, even if they were delivered at the local level by various intermediaries (Bennett, 2016). But this has never been the case for the larger financial support schemes which have largely been the purview of nationwide financial institutions such as the British Business Bank.^13^ In this sense, although the government sought to increase the supply of finance available, it was rarely driven by any estimation of regional need/demand and was left to its approved intermediaries to issue loans on a “first come, first served basis”, typically through its myriads of loan guarantee schemes.

The period we specifically investigate is from 2021 through to 2024, thus it includes the latter end of the Covid-19 pandemic and also the slow emergence from the pandemic period when many smaller firms faced a debt overhang problem as many SMEs had accumulated significant debts through the pandemic often due to a lack of precautionary savings (Cowling et al., 2020a, 2020b; Gourinchas et al., 2020). This problem led the UK government initiating new loan guarantee scheme, the RLS, which was designed specifically to facilitate lending so that smaller firms with a debt overhang problem would still be able to raise new loans to take advantage of new growth opportunities as they gradually emerged from the recessionary pandemic period.

In addition to this contribution in terms of policy guidance, the work also augments the direction of travel in terms of theorising around this contested analytical concept as being a multi-scaler rather than a mono-scaler one. Hitherto, by far the most dominant focal point for research on EE has been a mono-scaler perspective which some maintain is now increasingly being called into question by scholars (Brown et al., 2025). Increasingly, however EEs are viewed as being a “nested” concept where firms are located within wider economic geographies interconnected via a range of local, regional, national (and in some instances) international configurations (Spigel, 2022; Burström et al., 2024). Arguably, far from being disconnected from other spatial areas, EEs are increasingly being viewed from a multi-scaler theoretical vantage point in order to fully capture the full complexity of how space shapes entrepreneurial activity and opportunity (Schäfer et al., 2024; Theodoraki et al., 2023).

The paper empirically explores these spatial inter-relationships by testing whether or not what was happening to smaller firms within the wider NUTS 1 region in respect of RLS had any effect on what was happening at the second order spatial level, NUTS 2 (typically a county, or collection of smaller counties), and the third, and lowest order spatial level, NUTS 3 (typically a city or district within a city, or a part of a county). Our first set of analyses considered the higher order spatial effects on lower order spatial units. Here we found that NUTS 2 effects on NUTS 3 spatial units were generally more pronounced than the equivalent NUTS 1 effects, and subject to greater variation. It was also the case that NUTS 1 effect were greater and more pronounced on NUTS 2 than NUTS 3 spatial units. We also uncovered some unique findings relevant to the UK which is a highly diverse collection of nations (England, Scotland, Wales, and Northern Ireland), the latter three of which have devolved political powers. It also has London which has a unique status as an internationally important city and major hub for international finance (Brown et al., 2025).

Our findings in respect of the devolved nations, and also London, are that the higher order spatial effects are much larger and stronger on their lower order constituent spatial levels. This is not apparent for the rest of England. In this sense, there is a case for treating these places separately from England, or the rest of England outside of London when considering spatial ecosystems that are relevant to smaller firms. Merely considering all regions of the UK as being a comparable unit of spatial analysis for policy development, or indeed small firms ecosystem analysis, is not appropriate. Equally, it seems disingenuous to consider that a region that has more or less of something, here access to RLS loan funds, is a sign that small firms in all NUTS 3 areas within that region have the same level of access. Rather, we should look at the NUTS 2 level if we want a better understanding of what is happening at the NUTS 3 level if the smaller firm is the focus of our interest. Or as a second-best approximation, use NUTS 1 to help us understand more about NUTS 2.

However, if we consider the RLS scheme itself, then we have some evidence that the gap between spatial units of analysis is much closer, but still in favour of NUTS 2 helping explain more about NUTS 3 distribution of RLS loans. This suggests that at least some of the larger approved loan providers of RLS operate on a regional basis in terms of their allocation of loan funds. For example, a large UK national bank may allocate its SME loan funds annually across the UK regions according to some internal algorithm or other strategic decision-making process, which are then administered through their regional head offices. That said, the supply-side terms and conditions of such loans can often be spatially uneven both in terms of interest rates and levels of collateral sought by lenders (Bellucci, 2013; Cowling et al., 2020b) resulting in credit constraints for SMEs.

On balance, we are drawn to the conclusion that the most relevant spatial unit of analysis relevant to smaller firms is below the regional NUTS 1 level. In the same way as a Cornish person would consider themselves to be distinct from a person from Devon, small firms are intrinsically rooted in a local EEs where they do business and the wider region has little or no impact on what they do and their access to key resources. Close-knit spatial proximity matters. This suggests that in the UK, whilst regions, and especially super-regions, do not appear to have much relevance to smaller firms, but rather act as a political artefact for convenience in political discourse (Lee, 2017). The devolved nations are the exceptions, due to their greater political power and, to a lesser extent, their tax and spending autonomy. The theoretical ramifications of the work are clear.

## Conclusion

In this paper we set out to examine the most appropriate spatial level of analysis to help inform our understanding of how EEs function and operate in terms of the behaviour of their entrepreneurial constituents. In doing so the paper makes an important contribution to EE literature and one which has clear policy ramifications. Through the empirical lens of the UK SME support scheme, the RLS, we tested the relevance of different levels of EE geographical units such as, NUTS 1, NUTS 2 and NUTS 3. In the case of the UK, which is a diverse collection of nations (England, and three devolved nations, Scotland, Wales, and Northern Ireland), we found that the three devolved nations, and also London shows much larger and stronger higher order spatial effects on their lower order constituent spatial levels. On balance, we are drawn to the conclusion that the most relevant spatial unit of analysis relevant to smaller firms is below the regional NUTS 1 level. While entrepreneurial actors and resource providers operate across a number of spatial levels, firms are fundamentally rooted in their respective local spatial environment within which they are geographically located. This points towards the need for greater granular analysis (such as NUTS 3) towards helping our understanding of the full and intricate complexities of how EEs operate. This is crucial if tailored bespoke policies are to be orchestrated for local EEs. Our findings also support the theoretical arguments advanced in the literature (Brown et al., 2023) that primarily view EEs as “a complex system that emerges and evolves at local level” (Perugini, 2023, p.272).

The limitations of the present study are threefold. First, NUTS 1 regions in many other European countries do not generally encompass entire nations like they do in the UK which may limit the relevance of this study’s findings for other European countries. Relatedly, UK data availability at the lowest spatial level is very poor compared to that in continental Europe. Second, given the time period analysed (2021–2024) included the Covid-19 pandemic, sschemes like the RLS undoubtedly commenced at a unique time when firms were understandably keen to shore up their finances which made them more pre-disposed towards accessing external finance than they might have been otherwise before the Covid pandemic. In many respects this was a highly unique temporal situation which may have encouraged a higher level of borrowing within the SME community, especially in firms less pre-disposed to external borrowings than in ordinary circumstances. Finally, the authors wish to acknowledge the potential endogeneity issues of firm financing decisions which are likely to be impacted by the connection to a public loan guarantee scheme such as the RLS. Invariably, financing decisions are impacted by the presence or absence of a loan guarantee scheme and its parameters, particularly the level of guarantee.

We wish to end with a plea for further research to other EE scholars. As the EE literature develops, adopting a wider and more nuanced analytical lens will carry increasing currency as a mechanism for leveraging stronger insights into how geography shapes entrepreneurial behaviour across space at more granular spatial levels. The major problem for the researcher is that as we move down the spatial order to smaller geographies, the robustness and availability of data tends to dry up (Coad & Srhoj, 2023; Credit et al., 2018). There is great potential in the future for new forms of real-time data to help mitigate this problem and a concerted effort should be made to try and identify the solutions to these data gaps (see, for example, Rocha et al., 2022). Until then, researchers who continue to focus on large scale spatial territories such as NUTS 1 and NUTS 2 regions, will miss out on the richness and variation of what is happening to smaller firms in the more localised geographies where they predominantly inhabit, trade and operate.

## Data Availability

No datasets were generated or analysed during the current study.

## Appendix

See Table 5.

**Table 5:** Scotland RLS counts at NUTS 1, NUTS 2, and NUTS 3 levels

NUTS 1	Count	% of UK total	NUTS 2	Count	% of NUTS 1 total	NUTS 3	Count	% of NUTS 2 total
Scotland	1,734	5.46	Eastern Scotland	627	36.16	Angus and Dundee City	78	12.44
						City of Edinburgh	190	30.30
						Clackmannanshire and Fife	105	16.75
						East Lothian and Midlothian	80	12.76
						Falkirk	45	7.18
						Perth and Kinross, and Stirling	88	14.04
						West Lothian	41	6.54
			Highlands and Islands	145	8.36	Caithness and Sutherland, and Ross and Cromarty	25	17.24
						Inverness and Nairn, Moray and Badennoch and Strathspey	56	38.62
						Lochaber, Skye and Lochalsh, Arran and Cumbrae, and Argyll and Bute	38	26.21
						Na h-Eileanan Siar	7	4.83
						Orkney Islands	9	6.21
						Shetland Islands	10	6.90
			North Eastern Scotland	199	11.48	Aberdeen City and Aberdeenshire	199	100.00
			Southern Scotland	267	15.40	Dumfries and Galloway	35	13.11
						East Ayrshire and North Ayrshire mainland	51	19.10
						Scottish Borders	30	11.24
						South Ayrshire	39	14.61
						South Lanarkshire	112	41.95
			West Central Scotland	496	28.60	East Dunbartonshire, West Dunbartonshire, and Helensburgh and Lomond	41	8.27
						Glasgow City	260	52.42
						Inverclyde, East Renfrewshire, and Renfrewshire	91	18.35
						North Lanarkshire	104	20.97

## Footnotes

1 A recent search of Google Scholar in September 2024 using the term “entrepreneurial ecosystems” produced 329,000 results.

2 To be eligible firms must have a turnover of less than £45 m and the maximum loan size is less than £2 m.

3 https://www.british-business-bank.co.uk/finance-options/debt-finance/recovery-loan-scheme/recovery-loan-scheme-faqs

4 From 1 July 2024 the Recovery Loan Scheme has been extended and rebranded to the Growth Guarantee Scheme by the British Business Bank.

5 https://keep.eu/faq/nuts-what-is-it-how-does-this-relate-to-keep/#:~:text=NUTS1%3A%20Major%20socio%2Deconomic%20regions,Small%20regions%20for%20specific%20diagnoses.

6 North Wales is defined by the NP as covering: Wrexham, Rhyl, Colwyn Bay, Llandudno and Bangor.

7 To be eligible firms must have a turnover of less than £45 m and the maximum loan size is less than £2 m.

8 From 1 July 2024 the Recovery Loan Scheme has been extended and rebranded to the Growth Guarantee Scheme by the British Business Bank.

9 https://www.british-business-bank.co.uk/finance-options/debt-finance/recovery-loan-scheme/recovery-loan-scheme-faqs

10 Given that the dependent variables in our case are the count number of the loans issued at different spatial level, we assume this follows the Poisson distribution and the Poisson regression fits models of the number of occurrences (counts) of an event. As a robustness check, we also use different models and alternative dependent variables, and the results are consistent. Please see the additional checks in Sect. 4.4 and 4.5.

11 The result for the industry sector effect in Table 1 is omitted due to the length of the table. The full regression result will be available upon request.

12 See for example the regional cohesion funds that underpin the European Structural and Investment Funds that are financial tools governed by a common rulebook, set up to implement the regional policy of the European Union.

13 This was, in part, due to the historical failure of regional schemes such as the Regional Venture Capital Funds, although they are now back in political favour since the advent of the Nations and Regions Investment Funds.
